# Veterinary Experiences can Inform One Health Strategies for Animal Coronaviruses

**DOI:** 10.1007/s10393-021-01545-9

**Published:** 2021-09-20

**Authors:** Olivia S. K. Chan, Katriona C. F. Bradley, Alessandro Grioni, Susanna K. P. Lau, Wen-Ta Li, Ioannis Magouras, Tint Naing, Andrew Padula, Esther M. W. To, Hein Min Tun, Cedric Tutt, Patrick C. Y. Woo, Rebecca Bloch, Nathalie F. Mauroo

**Affiliations:** 1 LKS Faculty of Medicine, School of Public Health, Patrick Manson Building, The University of Hong Kong, Pokfulam, Hong Kong; 2 Tai Wai Small Animal and Exotic Hospital, G/F, Lap Wo Building, 69-75 Chik Shun St, Tai Wai, NT Hong Kong; 3 Fauna Conservation Department, Kadoorie Farm and Botanic Garden, Lam Kam Road, Tai Po, NT Hong Kong; 4 Department of Microbiology, The University of Hong Kong, Room 26, 19/F, Block T, Queen Mary Hospital, 102 Pokfulam Road, Hong Kong, Hong Kong; 5 Department of Pathology, Pangolin International Biomedical Consultant Ltd., Keelung, Taiwan; 6 Department of Infectious Diseases and Public Health, Jockey Club College of Veterinary Medicine and Life Sciences, City University of Hong Kong, Kowloon, Hong Kong; 7 Soares Avenue Paws and Claws Clinic, G/F No 29 - 33 Soares Avenue, Kowloon, Hong Kong; 8 Australian Venom Research Unit, Department of Pharmacology, Faculty of Medicine, University of Melbourne, Parkville, VIC 3010 Australia; 9 Agriculture, Fisheries and Conservation Department, The Government of Hong Kong Special Administrative Region, Room 509, Cheung Sha Wan Government Offices, 303 Cheung Sha Wan Road, Sham Shui Po, Kowloon, Hong Kong; 10 Cape Animal Dentistry Service, 78 Rosmead Avenue, Kenilworth, Cape Town, 7708 South Africa; 11 University of North Carolina at Chapel Hill, Chapel Hill, NC USA; 12 Hong Kong Wildlife Health Foundation, GPO Box 12585, Hong Kong, Hong Kong

**Keywords:** One Health, Coronavirus, Veterinary, COVID-19, Biosecurity, Vaccination, Wildlife, Spillover, Farm animals, Companion animals, Treatment

## Introduction

Coronaviruses in wild and domestic animals have a long history of spillover between species (Cui et al. 2019; Decaro and Lorusso 2020; Decaro et al. 2020). Severe acute respiratory syndrome (SARS-CoV) which emerged in 2002, followed by Middle East Respiratory Syndrome (MERS) in 2012, provided warnings of the capacity of coronaviruses to emerge from an animal source and cause severe adverse effects on both human health and economies (Osterhaus et al. 2020). Despite these warnings, SARS coronavirus 2 (SARS-CoV-2) crossed to humans in late 2019 and the scale of the coronavirus disease 2019 (COVID-19) pandemic and the damage it caused shook the world (Gruetzmacher et al. 2020; Morens et al. 2020).

Considerable veterinary experiences have been acquired over the past 70 years in studying and managing coronavirus infections in animals (Maclachlan and Dubovi 2010). This paper identifies some areas and experiences relevant to the prevention, control and treatment of COVID-19 and, more broadly, spillover of coronavirus from animals to humans and/or to other animals. Much has been published in these areas but the application of some of this information, from a One Health perspective, has not been explored fully or warrants reiteration. Therefore, in this paper, veterinarians from diverse backgrounds who work in small and exotic animal clinics, zoo and wildlife rehabilitation, government veterinary public health office, livestock veterinary services, academics and viral research laboratories have jointly reviewed coronavirus infection prevention and control strategies in animals and how these can be applied against coronavirus epidemics and pandemics. Aspects of coronavirus adaptation and spillover in pets, livestock and wild animals are discussed. Veterinary experiences in animal coronavirus epidemic biosecurity, along with pharmaceutical options such as vaccination programs and application of antiviral medications in domestic animals are reviewed. We conclude that veterinarians, as part of multidisciplinary teams, have been, and should continue to engage in coronavirus infection prevention and capacity building within the One Health framework.

## Veterinary Strategies Against Coronavirus Spillover Between Humans and Animals

Coronaviruses will likely continue to circulate and adapt between animals and humans (Brownlie and Sibley 2020). To date, seven human coronaviruses are known to originate in animals (Ye et al. 2020). Of these, three human pathogenic strains are most likely to have transmitted from or through animals: severe acute respiratory syndrome coronavirus (SARS-CoV), Middle East Respiratory Syndrome coronavirus (MERS-CoV) and SARS-CoV-2 (Singla et al. 2020). In the case of MERS-CoV, it is recognized that lack of consistent veterinary care is one of the drivers for spillover (Gossner et al. 2016; Zhu et al. 2019). Early on in the COVID-19 pandemic, reports of disease originating in seafood or reptiles became a theory of transmission. Godoy et al., veterinarian and marine biologists from Centro de Investigaciones Biológicas Aplicadas (CIBA), investigated and dispelled the theory of transmission through aquatic food animals (Godoy et al. 2021). Veterinarians have, and will continue, to act as first responders and to help diagnose animal coronavirus clinical cases to guide biosecurity thereafter. Coronavirus infections and the COVID-19 pandemic provide clear indications that veterinary, medical and research counterparts need to work together to understand the transmissibility of disease, be voices for prevention and close the gaps where and when coronavirus spillover occurs at the sylvatic, domestic animal and zoonotic interfaces.

At national and international levels, veterinary professionals, their representative associations and animal health regulatory bodies have played roles in protecting the public’s health during the COVID-19 pandemic due to their knowledge and first-hand experiences with animal coronaviruses. The United States Agency for International Development’s Emerging Pandemic Threats, the Pandemic Preparedness for Global Health Security (PREDICT) project, was managed by the School of Veterinary Medicine at the University of California, Davis, and was headed by wildlife veterinarians. Through this project, bio-surveillance of SARS-CoV-2 in animals and humans was conducted. The program underscores the significance of veterinarians and their capacities in coronavirus surveillance and control. Another example of the human-animal-ecosystem endeavor is the establishment of the World Organisation of Animal Health (OIE) “Ad Hoc Group on COVID-19 at the Human-Animal Interface.” The Group is led by Dr. William Karesh, president of OIE’s Working Group on Wildlife, and joined by collaborating groups to identify research priorities, assess and disseminate surveillance information, and provide guidance on veterinary services that are COVID-19-relevant in animal health and veterinary public health. In Ireland, veterinary epidemiologists work with medical and research colleagues of the National Public Health Emergency Team’s modeling advisory group to counter COVID-19 (Ferri and Lloyd-Evans 2021b) (https://www.gov.ie/en/publication/de1c30-national-public-health-emergency-team-nphet-for-covid-19-governance). These examples emphasize the need for wider mobilization of veterinary skills and expertise within national and international task forces in countering SARS-CoV-2 transmission and future epidemics.

In the advent of COVID-19 under the One Health framework, veterinarians, together with public health professionals, help draft guidelines to minimize bidirectional SAR-CoV-2 infections between animals and humans. The OIE working group on wildlife, the International Union for Conservation of Nature (IUCN) Species Survival Commission (SSC) and the Wildlife Health Specialist Group collectively produced “Guidelines for Working with Free-Ranging Wild Mammals”, among other risk assessments and preventive measures for care of captive wildlife. The IUCN SSC recommends that during the COVID-19 pandemic, no great ape reintroductions or translocations should take place (http://www.primate-sg.org/covid-19). As a precaution against COVID-19 spillover, the IUCN Group recommends biosecurity measures to prevent and control coronavirus transmission from humans to wild great apes, including guidelines on physical distancing, mandatory use of medical masks and various bans on visitation. Further examples of veterinarians researching, producing and following statements can be found at the Center for Disease Control, COVID-19 Safe Industry Plan for Zoos and Aquariums by the Australia Zoo and Aquarium Association, and OIE guidelines to prevent spillover of coronavirus around humans, terrestrial and aquatic animals (www.cdc.gov/coronavirus) (Mihindukulasuriya et al. 2008; Woo et al. 2014a; Franklin and Bevins 2020; McAloose et al. 2020). Under these circumstances, veterinary expertise provides strategies to navigate through the current COVID-19 pandemic and minimize passage of coronavirus infection (Fathke et al. 2020). It is recommended that these actions become standard in zoonotic and anthropozoonotic infectious disease management.

Animal coronavirus epidemics and COVID-19 pandemic represent only some of coronaviruses’ infection capacity (Morens et al. 2020). For rapid risk assessment, sustainable surveillance of coronaviruses is necessary. The surveillance of coronaviruses requires inter-disciplinary collaboration from teams of biologists, virologists, genomics scientists, veterinary and human medical professionals. Because veterinarians are familiar with animal disease patterns and differential diagnosis lists, screening or diagnostic tests, morbidity and mortality pattern and rates caused by different infectious diseases, husbandry and sampling flaws, the profession can help set up animal coronavirus surveillance network particularly in hotspot countries (Morens et al. 2020). This system should be built around animal coronavirus surveillance for One Health professionals and made publicly available. It should contain clear sampling protocol similar to that of Mustelids SARS-CoV-2 surveillance platform requested by European Commission and recommended by European Food Safety Authority, with electronic and notification platform similar to that of OIE-World Animal Health Information System (WAHIS) (Foddai et al. 2020; Bhatia 2020).

## Animal Coronavirus Spillover and Adaptation

The history of coronavirus spillover, mutation and adaptation is well documented and will not be described in detail here, although the precise origin of viruses has not always been determined (Decaro et al. 2020; Lawal and Onoja 2020). From a One Health and veterinary perspective, coronavirus spillover is best addressed at the source, in order to reduce opportunities for exposure to animals or humans likely to be infected with coronaviruses (Epstein et al. 2020; Morens et al. 2020).

Fourteen months after emergence as a pandemic human pathogen, much still needs to be learned about the events leading to spillover of SARS-CoV-2 to humans. It has been proposed that SARS-CoV-2 originated in bats but the exact pathway from bats to humans or the site where this occurred had not been determined (Lau et al. 2020). In the quest to identify possible intermediate hosts, SARS-CoV-2 related virus was identified in archived tissue samples from confiscated Malayan pangolin (Lam et al. 2020; Zhang et al. 2020). However these viruses were not detected in animals in Malaysia, suggesting they were unlikely to be reservoir hosts for SARS-CoV-2, at least in some parts of their natural habitat (Lee et al. 2020). SARS-CoV-2-like virus was detected in pangolins in China by veterinarians at Wildlife Rescue Center at the South China Agricultural University. This finding further demonstrates pangolins can be infected further along the trade network, posing potential risks when they enter markets and mix with other species, including humans (Xiao et al. 2020; Sivasundaram 2020; Frutos et al. 2020b; Afrasiabi et al. 2020).

Ample opportunities exist for direct or indirect exposure of humans to bats capable of shedding coronavirus (Li et al. 2019; Epstein et al. 2020). To reduce the risk of exposure, changes in behavior in rural communities can be applied, as has been demonstrated by measures used to reduce direct and indirect contact (Nahar et al. 2017; Li et al. 2020). Studies conducted on sale networks of bats to markets in Sulawesi in Indonesia suggested live bats are not sold in markets, indicating it is the sites where bats are killed and handled prior to cooking, that need to be targeted for interventions (Latinne et al. 2020). Further findings indicate strategic changes in planning and behavior in farming and rural communities are critical to reduce contact with bats, as has been demonstrated with measures used to reduce indirect contact with pteropodid bats (the host of Nipah virus) (Pulliam et al. 2012; Rabinowitz and Conti 2013; Nahar et al. 2017). A Nipah virus outbreak investigation in Malaysia highlights the importance of better communication among medical and veterinary health professionals (Kulkarni et al. 2013). In the outbreak, without knowledge of increased pig mortality, human Nipah virus cases were misdiagnosed as Japanese encephalitis (Mackenzie 2009). The communication of information on pig mortality and veterinary clinical findings could have expedited Nipah virus detection. Another example that supports One Health collaborative research was conducted by veterinarians, virology researchers and the OIE Collaborating Centre for Diseases at the Animal/Human Interface. Based on coronavirus transmission findings among farms with various husbandry practices, the authors made biosecurity recommendation against pig-bat contact (Leopardi et al. 2021). Such recommendations, when adopted, can help prevent or remove animals from being intermediate or magnifying hosts of SARS-CoV-2 or other coronavirus transmission. To continue support One Health strategies, it is important to continue traditional veterinary roles and responsibilities to diagnose and prevent animal diseases, as well as strengthen and develop veterinary expertise in research, epidemiology, policy, surveillance and leadership (Fathke et al. 2020; Sofi et al. 2021; Ferri and Lloyd-Evans 2021a; Osburn et al. 2009; Cipolla et al. 2015).

Animal coronavirus spillover does not always result in immediate adaptation or mutation to adapt to a new host nor do coronaviruses have equal capacity to transmit to different animal species. Some appear to be dead-end hosts, as has been observed to date with zooanthroponotic transmission of SARS-CoV-2 to dogs (Temmam et al. 2020). Based on precedent experience with SARS where human-to-pet animal transmission was documented, veterinarians and epidemiologists at the Department of Agriculture and Fisheries in the Hong Kong Special Administrative Region investigated human-to-dog transmission and identified SARS-CoV-2 RNA and antibodies in affected pet dogs (Sit et al. 2020). So far, transmission outside of households remains inconclusive (Temmam et al. 2020; Colitti et al.; Vlasova et al. 2021). In swine farms and clinical practices, veterinary knowledge indicates coronaviruses in pigs (such as hemagglutinating encephalomyelitis virus), cats (Feline Enteric Coronavirus (FeCV), the causative agent of Feline Infectious Peritonitis (FIP)), and dogs (Canine Coronavirus types I & II (CCV)) have a long history of infection in a single species suggesting spillover to humans or other animals does not occur or occurs rarely (Fenner et al. 2014). Among camels, three MERS-coronavirus strains circulate in dromedaries, each causes mild respiratory and diarrhea symptoms in camels. Yet only one of the three strains has spilled over to cause MERS in human (Wünschmann et al. 2002; Woo et al. 2014b; Omrani and Shalhoub 2015; Sabir et al. 2016).

Veterinarians and researchers have identified animal coronaviruses that can adapt to new hosts and also change over time within an established host. Examples of these processes in farmed and wild animals abound (Frutos et al. 2020a; Leroy et al. 2020; Bartlett et al. 2021; Halfmann et al. 2020). Porcine Epidemic Diarrhea Virus (PEDV) was first reported in pigs in the 1970s in the United Kingdom. Subsequently highly virulent PEDV strain emerged in Asia and the United States (Stevenson et al. 2013; Wang et al. 2019; Vlasova et al. 2020; Lee 2015). Domestic and wild felids are also susceptible to SARS-CoV-2 (Bartlett et al. 2021; Leroy et al. 2020). Experimentally, transmission by contact between infected and uninfected cats has occurred, suggesting the virus is relatively well adapted for transmission with this species (Halfmann et al. 2020). However, spillover does not only occur within species, as veterinarians diagnose animal coronavirus infection that appear to be well adapted to their host and can, on occasion, spillover to other species. In farms, porcine coronavirus is found to spillover from wild birds to farmed pigs (Wang et al. 2019; Vlasova et al. 2020). In the wild, coronavirus that spillover from asymptomatic bats can lead to swine acute diarrhea syndrome coronavirus (SADS-CoV) (Zhou et al. 2018). In SADS-CoV, the knowledge and diagnosis of swine acute diarrhea syndrome are important, especially with recent experimentation evidence that SADS-CoV can infect human cells (Edwards et al. 2020). Humans are known to have been infected with MERS virus from handling infected camels that were asymptomatic (Azhar et al. 2014).

Animal coronavirus spillover occurs in all directions between the human-sylvatic and human-domesticated animal interfaces (Olival et al. 2020; Gossner et al. 2016; Wardeh et al. 2021). As part of the preventive public health, veterinary experiences help devise ways to minimize animal-human interaction, or risk thereof, among wild, farmed, pet or zoo animals. In terms of zoonotic coronavirus, being closely related genetically, such as that between humans and non–human primates, appears to increase the likelihood of pathogen transmission between species (Davies and Pedersen 2008). Viral reverse zoonoses associated with high morbidity and mortality is a threat among great apes. In 2018, an example in Cote d’Ivoire indicates human coronavirus OC43 was transmitted to captive chimpanzees (Patrono et al. 2018). Documented respiratory outbreaks results from SARS-CoV-2 are associated with death in chimpanzee and gorilla populations with possibility to circulate back to human (Ghai et al. 2021). Another particular concern is the release of wild animals, which if infected with SARS-CoV-2, could establish infections in the wild (Franklin and Bevins 2020; de Sadeleer and Godfroid 2020). For domestic pet owners, especially those infected with SARS-CoV-2, veterinary recommendations have been developed to prevent zooanthroponotic transmission (OIE 2020). Reverse zoonoses are seen in other species besides great apes. In farm animals, it is shown that minks are susceptible to infection with SARS-CoV-2 given efficient transmission that occurred in this species on farms in a number of countries (Molenaar et al. 2020). Likely spillback of virus from farmed mink to humans has already been observed (Opriessnig and Huang 2020; Munnink et al. 2020). Genetic adaptive changes in SARS-CoV-2 in mink represent a potentially important zoonotic and anthropozoonotic risks (Manes et al. 2020; Munnink et al. 2020; Colitti et al.; Koopmans 2021; Boklund et al. 2021) and guidance has been developed for mink farms (USDA 2020; Molenaar et al. 2020). Specific OIE guidelines have been produced for different taxa (great apes, bats, felids, mustelids) to prevent spillover (oie.int/en/global-theme/covid-19). Concerns of strain mutation and spillover behoove veterinary profession to continue monitor, survey and update guidelines on coronaviruses (He et al. 2021; Plante et al. 2021; Khamassi Khbou et al. 2021).

## Biosecurity Strategies, Disease Prevention and Virus Elimination

In the advent of the COVID-19 pandemic, although human vaccinations are widely promulgated, given the risk of spillover, adaptation and mutation, it is worthwhile to iterate some of the biosecurity lessons learned from animal coronaviruses (Albert et al. 2021). Most of these protocols aim to prevent entry of pathogens (referred to as bioexclusion or external biosecurity) and to prevent their onward transmission in case bioexclusion fails (referred to as biocontainment or internal biosecurity) (FAO 2010) (Brennan and Christley 2012; Mendez et al. 2014; Gray and Merchant 2018). In terms of clinical prevention against animal coronavirus, veterinary and clinic management teams formulate and implement sanitation practices such as strict cleaning and disinfection protocols used for PEDV and heat treatment in kennels against feline and canine coronaviruses outbreaks (Pratelli 2007; Bowman et al. 2015; Kumar 2020). For FeCV, biocontainment measures including good hygiene, use of disposable bedding and designated feeding utensils are the mainstays of coronavirus transmission prevention and veterinary clinic protocols (Hosie et al. 2021; Addie 2019). Another example is the Norwegian control program to combat bovine coronavirus (BoCV) which relies on engaging the cattle industry and veterinarians to enforce biosecurity barriers and regulate live animal trade to reduce direct and indirect coronavirus transmission (Stokstad et al. 2020; Murray et al. 2016). Similar biosecurity measures have been applied in human health but there are areas where experiences and expertise from animal health could have been applied at the initial outbreak of COVID-19. Serial testing of workers was recommended to apply in the pandemic to high risk work places such as aged care facilities and quarantine centers in July, 2020 (Sims 2020; Sumption et al. 2020). In November 2020, serological assay and antigen serial testing were established among mink farm workers and their household in Ireland subsequent to COVID-19 outbreak in Denmark’s mink farms (Munnink et al. 2021; Hammer et al. 2021). To provide a comprehensive disease risk management framework between human and animals, veterinarians and doctors must converge to bring together biosecurity practices needed for human, animal, plant and environmental health (Hulme 2020).

Other bioexclusion measures used in the veterinary field include restrictions or bans on imports of animals or animal products from places where particular diseases are present, as well as pre- and post-entry quarantine and testing. When applied appropriately, these measures allow high health status countries to maintain freedom from infection with certain pathogens while retaining trading in animals and animal products. For example, many countries remain free from a range of coronaviral infections, including virulent PEDV, as a result of biosecurity measures, despite importing pig products (Kim et al. 2017). Two months into COVID-19 pandemic, veterinarian recommended bioexclusion measures for COVID-19 in humans that include limiting entry of travelers from places where the disease is poorly contained, use of suitable PPE by all in transit (Per International Air Transport Association (IATA), PPE might include: face protection, goggles and mask or face shield, gloves, gown or coverall), and enhanced pre-arrival and post-arrival testing and quarantine (Sims 2020). Strict bioexclusion approaches on passenger arrivals have managed to limit the number of imported cases (e.g., Vietnam) and this has facilitated efforts to eliminate community transmission in places such as Australia and New Zealand at the initial phase of the pandemic (Baker et al. 2020a, b; Summers et al. 2020; Heywood and Macintyre 2020).

Fomites play an important role in the transmission of animal coronaviruses. Previous studies have shown animal coronaviruses can survive on fomites for several days and PEDV can survive in sewage lagoons for several months, including a winter season (Tun et al. 2016). Further evidence suggests persistence of SARS-CoV-2 on fomites is a potential biosecurity concern for humans (Riddell et al. 2020). However, fomite transfer is not regarded as being as important as airborne spread for SARS-CoV-2, especially in places where human infections are poorly controlled. Nevertheless, droplet contamination of surfaces can occur and SARS-CoV-2 can survive for extended periods on frozen and chilled produce and packaging (Ji et al. 2021). An outbreak in Beijing may be linked to exposure of market workers to contaminated, imported, frozen salmon (Pang et al. 2020). Therefore, precautions should still be taken when handling potentially contaminated produce, especially in places where community transmission has been eliminated.

Disease control and elimination depend on appropriate surveillance systems. “Pre-outbreak surveillance” strategies in wildlife and routine farm and market surveillance are critical in identifying threats and designing programs to reduce the risk of zoonotic disease spillover (Daszak et al. 2020). In Indonesia, the PREDICT project trains local Payangan veterinarian from the Indonesia Department of Animal Health and has setup a surveillance system among local pig farms against PEDV (http://www.fao.org/3/i7778e/i7778e.pdf). In addition, the US Centers for Disease Control and Prevention (US-CDC) co-funded the development of a Field Epidemiology Training Programme for Veterinarians (FETPV) in Indonesia in 2017 which helps Indonesia to gatekeep against emerging animal coronavirus epidemics. Current surveillance systems were found to be inadequate in a number of countries when COVID-19 emerged. Surveillance for infectious agents in farmed animals and wildlife is both underfunded and often inconsistent, with reduced interest in disease during inter-epidemic periods. There have been strong calls for enhanced wild animal surveillance to determine the extent and nature of the threats posed and for appropriate action to be taken (Watsa 2020).

## Coronavirus Treatment and Vaccination in Animals

COVID-19 has called for an urgent demand for safe and effective pharmaceutical options for treatment and prevention against coronaviruses (Sanders et al. 2020). Treatments against coronaviruses in diseased animals are well documented and will be described only briefly here (Fenner et al. 2014). In the last decade, medication for FIP has included at least three new generation and two traditional drugs (Legendre et al. 2017; Pedersen et al. 2018, 2019; Addie et al. 2020). Remdesivir, a prodrug that inhibits RNA viral polymerase, controls FIP symptoms in several studies, with side effects of pain, ulceration and scarring at injection sites (Murphy et al. 2018; Pedersen et al. 2019). FIP infected cats treated with antiprotease inhibitors, which target coronavirus’s 3C-like protease, showed improvement but relapsed in one to seven weeks (Pedersen et al. 2018). This inhibitor is related to the drug lopinavir, which is currently being tested in humans with severe COVID-19 (Stower 2020). In human COVID-19 cases, oral lopinavir-ritonavir have not reduced mortality (Cao et al. 2020). A product containing remdesivir has been approved for use by the Food and Drug Administration (USA FDA) based on studies indicating a shorter time to recovery and better odds of clinical improvement in human patients infected with SARS-CoV-2 at day 15 (Beigel et al. 2020; WHO Solidarity Trial Results 2020).

Vaccination against coronaviruses in animals is a possible, yet imperfect, avenue to prevent disease caused by coronaviruses and has been considered in depth elsewhere (Tizard 2020). The main coronavirus vaccines used in animals are against infectious bronchitis virus (IBV) in chickens, Transmissible gastroenteritis virus (TGEV) and PEDV in pigs (Tizard 2020). Vaccines have also been used to assist in prevention of FeCV, bovine and canine coronavirus infections with the latter two usually forming part of multi-agent vaccines when deployed. Results of vaccination against IBV show that immunity can be developed using vaccines against homologous strains but also demonstrate that viral mutations potentially result in antigenic changes which present a challenge for vaccination programs (Cook et al. 2012). Most commercial chicken flocks are vaccinated and usually require booster doses of vaccine. Most vaccination programs utilize live, attenuated vaccines with booster doses of live or killed virus vaccines in long-lived birds such as layers and breeders. Due to the scale of chicken production, mass vaccination is applied using spray, drinking water, or by eyedrops in day-old chicks with an attenuated virus (Jackwood et al. 2010). Programs in different geographic areas and using a range of vaccine antigens resulted in 60–90% vaccine effectiveness (Cavanagh 2003; Grgić et al. 2009). Some of the problems with IBV vaccination are overcome using multivalent vaccines and rigorous vaccination programs, but control of IBV infection is a constant challenge for the industry (Martin et al. 2007; Grgić et al. 2009). Part of the incomplete vaccine coverage is due to the existence of different serotypes and the lack of cross protection against heterologous strains (Jordan 2017). Reversion to virulence of attenuated strains has been recorded, as has recombination between vaccine and field strains (Bande et al. 2015; Ren et al. 2020).

In pig industry, vaccines for PEDV, especially for the highly virulent strains which emerged in the past decade have not been particularly effective and novel vaccines based on new technologies are being applied. These include a replicon particle vaccine used in the US, vector vaccines and vaccines with enhanced adjuvants (Kim et al. 2016). Coronavirus vaccines are also used to confer passive immunity to offspring or reduce zoonotic potential. In swine, vaccines against TGEV and PEDV are aimed at breeding animals to provide passive immunity to offspring born to immune sows (Gerdts and Zakhartchouk 2017). In cattle, vaccines are also administered to breeding cows to allow transfer of immunity via colostrum (Crouch et al. 2000). In camelids, experimental MERS coronavirus spike protein subunit vaccines confer complete protection in alpacas and delays viral shedding in dromedary camels in naive animals thereby reducing zoonotic transmission (Adney et al. 2019). Yet despite these developments, vaccination against MERS in camels has not yet been taken up as a One Health solution due to lack of licensure (Shen et al. 2019). Even with other severe zoonotic diseases such as Hendra virus in horses, for which vaccines have been developed, uptake has been less than ideal due to owners’ perceptions of vaccine safety, cost and effectiveness (Manyweathers et al. 2017). Vaccines could be considered for use in wild animal populations against viruses with pandemic potential but a number of hurdles need to be overcome before this becomes a reality (Nuismer and Bull 2020). Rat coronaviruses have high strain variation, and cross-protection by vaccination is low, thus allowing viral shedding and recurrence of milder clinical signs, suggesting vaccination of wild rat populations against coronaviruses is not likely to be feasible (Bihun and Percy 1994). In small animals, vaccines have been developed for dogs and cats but are not recommended by the World Small Animal Veterinary Association. One of the profession’s concerns about these vaccines is the potential for the vaccine to enhance disease. It was noted that with high virulent challenge by FIP vaccine, some vaccinated cats develop more acute disease with more severe clinical signs, and a greater mortality compared to unvaccinated control cats (Scott 1999). In farm and wild animals, another concern is spillover that may result from the use of live virus vaccine strains, as has been seen with IBVs in wild birds (Miłek and Blicharz-Domańska 2018). While none of these vaccines will be adopted for use in humans, many of the lessons learned from vaccination against animal coronaviruses are still pertinent.

As SARS-CoV-2 continues to spread and mutate, development of coronavirus vaccines will likely be an ongoing process. Not to be directly extrapolated, experiences from animal vaccine development can provide experimental and clinical case-studies and models for humans (Koyama et al. 2020; Grubaugh et al. 2020; Deb et al. 2020). Bovine coronavirus (BCV) and PEDV vaccination programs illustrate the transfer of maternal antibodies from vaccinated mothers to their newborns, which plays a major role in protective immunity. In the poultry industry, some producers give more than one vaccine serotype to increase the breadth of protection in their flocks because strain variation is a challenge in poultry IBV control (Jordan 2017; Bok et al. 2018). In the meantime, there is no data on the safety or efficacy of mixing COVID-19 vaccines in human (Del Rio and Malani 2021). As a reflection on precautionary measures, FIP vaccine with low-virus dose protects kittens, while high-virus dose offsets vaccine protection and induces accelerated FIP (Fehr et al. 1997; Vennema et al. 1990).

## Ecosystem and Environmental Considerations

Within the One Health framework, human actions play a major role in disease ecology of coronaviruses and in biodiversity conservation (Rabinowitz and Conti 2013). As noted in Nipah virus epidemics, livestock expansion, habitat destruction and fragmentation increase spillover risks (Looi and Chua 2007; Walsh et al. 2017). In addition, over-exploitation and consumption of threatened species increase contact between those species and humans, predisposes wild animals to immunosuppression and viral challenge, leads to population decline in those animal species and biodiversity loss, which in some situations are the factors for increased spillover of viral diseases (Keesing et al. 2010; Johnson et al. 2020; Roe et al. 2020; Magouras et al. 2020). Effective prevention and control of transmissible animal diseases, including zoonoses surveillance is a core task of veterinary services of each OIE Member Country (de Sadeleer and Godfroid 2020).

Animal SARS-CoV-2 investigations drew attention to the extent of a largely unregulated and often illegal trade, in pangolins and numerous other threatened species. Compared to domestic species, wild animals transported internationally undergo at best limited viral pathogen screening, and at worst, none as the illegal wildlife trade has a total absence of monitoring (Halabowski and Rzymski 2020). For instance, thousands of pangolins were traded illegally in the past decade for human consumption and traditional medicine (Cheng et al. 2017). The unmonitored international transfer of pathogens with bushmeat evades veterinary public health inspection and threatens human, animal and environmental health worldwide (Can et al. 2019; van Roon et al. 2019). Veterinary epidemiology is key to measuring disease frequencies, identifying risk factors and implementing biosecurity programs (Robertson 2020). In addition, veterinarians are essential for wildlife management and research, disease surveillance and prevention, training and education, as well as the maintenance of wildlife health as part of multi-disciplinary wildlife management teams (Kock 1996; Gortázar and de la Fuente 2020; Robertson 2020).

Environmental disturbances are primers for emerging infectious diseases (EID), and in turn, EID prove to be very costly, both monetarily and environmentally (Daszak et al. 2001). Changes in land use, including expansion of livestock farming leading to encroachment into wildlife habitat, increase and modify interactions at human-wildlife-domestic animal interfaces, resulting in spillover. Concurrently, there is insufficient information to understand coronavirus transmission in wild animals. For example, only limited experimental information is available concerning pathological changes in masked palm civets as intermediate hosts in SARS-CoV (Xiao et al. 2008). The loss of surveillance interest during inter-epidemic periods reflects the difficulty to sustain coronavirus investigation—thus it is important to expand veterinary engagement, maximize clinical findings, molecular and serological results for detection of viral transmission and pathologies in wild animals. If not already in place, veterinarians should routinely perform comprehensive and systematic post-mortem and histopathological examinations in wild animals for readiness of wild animal host identification in times of EID outbreaks. Additionally, structure and resources commitment is needed so that symptomatic and asymptomatic wild animals can be sampled in wildlife rehabilitation centers.

## Conclusion

The experiences gained over the past 70 years in managing diseases caused by coronaviruses (and other animal diseases) have provided veterinarians with insights into ways to address the COVID-19 pandemic and also to prevent future spillovers. Transmission of coronaviruses between species and adaptive changes have occurred on many occasions and appear to have accelerated in the past 20 years. Anthropogenic factors such as biodiversity loss, environmental degradation and inappropriate farming and selling practices, though contending at contribution levels, must be investigated and addressed to reduce and mitigate future spillover and pandemics (Roche et al. 2020). Food animal production, selling and consumption should be undertaken in a way that poses minimal threat to animal health, public health and environmental health. Humans can no longer afford to continue with “business as usual”. We have to reduce the likelihood of spillover of coronaviruses and other animal pathogens. COVID-19 has to be used as a catalyst for transformations covering areas such as the location of farms, biosecurity measures along the food value chain, and poorly controlled trade in wild animals and their products. Management of coronavirus diseases in animals has depended more on biosecurity measures than vaccination, given the limited efficacy of vaccines and treatments against many of these diseases. Experiences with vaccination of birds against avian coronavirus have demonstrated the difficulties in obtaining protection against antigenic variant heterologous strains and also the potential for recombination between live virus vaccines and field viruses.

Veterinarians’ expertise and knowledge, particularly experiences in epidemics that involve multiple hosts, primary, secondary and evolving pathogens, can improve One Health approach to this disease and other emerging diseases (McNamara et al. 2020). As indicated in managing coronavirus spillover and COVID-19 pandemics, veterinarians can play a larger role in public health decisions, as an individual, or through professional associations and animal health regulatory bodies both at national and transboundary levels. As veterinarians oversee pet, livestock and wild animal health in a One Health framework, collaboration among ecologists, medical and veterinary professionals can strengthen and become common practice in zoonotic disease surveillance and management (Sofi et al. 2021; Cunningham et al. 2017). As part of a multi-disciplinary team, veterinarians should be more active in non-traditional fields such as research, academia, and policymaking, interacting with other professions to advocate for ecosystem health, as well as animal health.

## References

[CR1] Addie DD (2019). Feline infectious peritonitis: answers to frequently asked questions concerning FIP and coronavirus. Veterinary Nursing Journal.

[CR2] Addie DD, Curran S, Bellini F, Crowe B, Sheehan E, Ukrainchuk L, Decaro N (2020). Oral Mutian® X stopped faecal feline coronavirus shedding by naturally infected cats. Research in Veterinary Science.

[CR3] Adney DR, Wang L, Van Doremalen N, Shi W, Zhang Y, Kong W-P, Miller MR, Bushmaker T, Scott D, de Wit E (2019). Efficacy of an adjuvanted Middle East respiratory syndrome coronavirus spike protein vaccine in dromedary camels and alpacas. Viruses.

[CR4] Afrasiabi A, Alinejad-Rokny H, Lovell N, Xu Z, Ebrahimi D (2020) Evidence for ZAP-independent CpG reduction in SARS-CoV-2 genome, and pangolin coronavirus origin of 5’UTR. *bioRxiv*

[CR5] Albert C, Baez A, Rutland J (2021) Human security as biosecurity: Reconceptualizing national security threats in the time of COVID-19. *Politics and the Life Sciences* 1–23 10.1017/pls.2021.1 PMC7902155 33949836

[CR6] Azhar EI, El-Kafrawy SA, Farraj SA, Hassan AM, Al-Saeed MS, Hashem AM, Madani TA (2014). Evidence for camel-to-human transmission of MERS coronavirus. New England Journal of Medicine.

[CR7] Baker MG, Kvalsvig A, Verrall AJ, Wellington N (2020). New Zealand’s COVID-19 elimination strategy. Med J Aust.

[CR8] Baker MG, Wilson N, Blakely T (2020) Elimination could be the optimal response strategy for covid-19 and other emerging pandemic diseases. *bmj* 371 10.1136/bmj.m4907 33561814

[CR9] Bande F, Arshad SS, Bejo MH, Moeini H, Omar AR (2015) Progress and challenges toward the development of vaccines against avian infectious bronchitis. *Journal of Immunology Research* 10.1155/2015/424860 PMC4411447 25954763

[CR10] Bartlett SL, Diel DG, Wang L, Zec S, Laverack M, Martins M, Caserta LC, Killian ML, Terio K, Olmstead C (2021). 'SARS-CoV-2 Infection And Longitudinal Fecal Screening In Malayan Tigers (Panthera tigris jacksoni), Amur Tigers (Panthera tigris altaica), And African Lions (Panthera leo krugeri) At The Bronx Zoo, New York, USA'. Journal of Zoo and Wildlife Medicine.

[CR11] Beigel JH, Tomashek KM, Dodd LE, Mehta AK, Zingman BS, Kalil AC, Hohmann E, Chu HY, Luetkemeyer A, Kline S (2020). Remdesivir for the treatment of Covid-19. New England Journal of Medicine.

[CR12] Bhatia R (2020). Need for integrated surveillance at human-animal interface for rapid detection & response to emerging coronavirus infections using One Health approach. The Indian Journal of Medical Research.

[CR13] Bihun CG, Percy DH (1994). Coronavirus infections in the laboratory rat: degree of cross protection following immunization with a heterologous strain. Canadian Journal of Veterinary Research.

[CR14] Bok M, Alassia M, Frank F, Vega CG, Wigdorovitz A, Parreno V (2018). Passive immunity to control Bovine coronavirus diarrhea in a dairy herd in Argentina. Revista Argentina De Microbiologia.

[CR15] Boklund A, Gortázar C, Pasquali P, Roberts H, Nielsen SS, Stahl K, Stegeman A, Baldinelli F, Broglia A, Van Der Stede Y, Adlhoch C, Alm E, Melidou A, Mirinaviciute G (2021). Monitoring of SARS-CoV-2 infection in mustelids. EFSA J.

[CR16] Bowman AS, Krogwold RA, Price T, Davis M, Moeller SJ (2015). Investigating the introduction of porcine epidemic diarrhea virus into an Ohio swine operation. BMC Veterinary Research.

[CR17] Brennan ML, Christley RM (2012). Biosecurity on cattle farms: a study in north-west England. PloS One.

[CR18] Brownlie J, Sibley D (2020). What can animal coronaviruses tell us about emerging human coronaviruses?. The Veterinary Record.

[CR19] Can ÖE, D'Cruze N, Macdonald DW (2019). Dealing in deadly pathogens: Taking stock of the legal trade in live wildlife and potential risks to human health. Global Ecology and Conservation.

[CR20] Cao B, Wang Y, Wen D, Liu W, Wang J, Fan G, Ruan L, Song B, Cai Y, Wei M (2020). A trial of lopinavir–ritonavir in adults hospitalized with severe Covid-19. New England Journal of Medicine.

[CR21] Cavanagh D (2003). Severe acute respiratory syndrome vaccine development: experiences of vaccination against avian infectious bronchitis coronavirus. Avian Pathology.

[CR22] Cheng W, Xing S, Bonebrake TC (2017). Recent pangolin seizures in China reveal priority areas for intervention. Conservation Letters.

[CR23] Cipolla M, Bonizzi L, Zecconi A (2015). From “One Health” to “One Communication”: the contribution of communication in veterinary medicine to public health. Veterinary Sciences.

[CR24] Colitti B, L Bertolotti, A Mannelli, G Ferrara, A Vercelli, A Grassi, C Trentin, S Paltrinieri, C Nogarol, and N Decaro. 'Early Release-Cross-Sectional Serosurvey of Companion Animals Housed with SARS-CoV-2–Infected Owners, Italy'. 10.3201/eid2707.203314 PMC8237875 33974535

[CR25] Cook JKA, Jackwood M, Jones RC (2012). The long view: 40 years of infectious bronchitis research. Avian Pathology.

[CR26] Crouch CF, Oliver S, Hearle DC, Buckley A, Chapman AJ, Francis MJ (2000). Lactogenic immunity following vaccination of cattle with bovine coronavirus. Vaccine.

[CR27] Cui J, Li F, Shi Z-L (2019). Origin and evolution of pathogenic coronaviruses. Nature Reviews Microbiology.

[CR28] Cunningham AA, Daszak P, Wood JLN (2017). One Health, emerging infectious diseases and wildlife: two decades of progress?. Philosophical Transactions of the Royal Society b: Biological Sciences.

[CR29] Daszak P, Cunningham AA, Hyatt AD (2001). Anthropogenic environmental change and the emergence of infectious diseases in wildlife. Acta Tropica.

[CR30] Daszak P, Olival KJ, Li H (2020). A strategy to prevent future epidemics similar to the 2019-nCoV outbreak.

[CR31] Davies TJ, Pedersen AB (2008). Phylogeny and geography predict pathogen community similarity in wild primates and humans. Proceedings of the Royal Society b: Biological Sciences.

[CR32] de Sadeleer N, Godfroid J (2020). 'The Story behind COVID-19: Animal Diseases at the Crossroads of Wildlife, Livestock and Human Health'. European Journal of Risk Regulation.

[CR33] Deb B, Shah H, Goel S (2020). Current global vaccine and drug efforts against COVID-19: Pros and cons of bypassing animal trials. Journal of Biosciences.

[CR34] Decaro N, Lorusso A (2020). Novel human coronavirus (SARS-CoV-2): A lesson from animal coronaviruses. Veterinary Microbiology.

[CR35] Decaro N, Martella V, Saif LJ, Buonavoglia C (2020). COVID-19 from veterinary medicine and one health perspectives: What animal coronaviruses have taught us. Research in Veterinary Science.

[CR36] Del Rio C, Malani P (2021). COVID-19 in 2021—Continuing Uncertainty. JAMA.

[CR37] Edwards CE, Yount BL, Graham RL, Leist SR, Hou YJ, Dinnon KH, Sims AC, Swanstrom J, Gully K, Scobey TD (2020). Swine acute diarrhea syndrome coronavirus replication in primary human cells reveals potential susceptibility to infection. Proceedings of the National Academy of Sciences.

[CR38] Epstein JH, Anthony SJ, Islam A, Kilpatrick AM, Khan SA, Balkey MD, Ross N, Smith I, Zambrana-Torrelio C, Tao Y (2020). Nipah virus dynamics in bats and implications for spillover to humans. Proceedings of the National Academy of Sciences.

[CR39] Fathke RL, Rao S, Salman M (2020). The COVID-19 pandemic: A time for veterinary leadership in one health.

[CR40] Fehr D, Holznagel E, Bolla S, Hauser B, Herrewegh AAPM, Horzinek MC, Lutz H (1997). Placebo-controlled evaluation of a modified life virus vaccine against feline infectious peritonitis: safety and efficacy under field conditions. Vaccine.

[CR41] Fenner FJ, Bachmann PA, Gibbs EPJ (2014). Veterinary virology.

[CR42] Ferri M, Lloyd-Evans M (2021a) The contribution of veterinary public health to the management of the COVID-19 pandemic from a one health perspective. *One Health* 100230 10.1016/j.onehlt.2021.100230 PMC7912361 33681446

[CR43] Ferri M, Lloyd-Evans M (2021). The contribution of veterinary public health to the management of the COVID-19 pandemic from a One Health perspective. One Health (amsterdam, Netherlands).

[CR44] Foddai A, Lindberg A, Lubroth J, Ellis-Iversen J (2020). Surveillance to improve evidence for community control decisions during the COVID-19 pandemic–opening the animal epidemic toolbox for public health.

[CR45] Franklin AB, Bevins SN (2020). Spillover of SARS-CoV-2 into novel wild hosts in North America: A conceptual model for perpetuation of the pathogen. Science of the Total Environment.

[CR46] Frutos R, Lopez Roig M, Serra-Cobo J, Devaux CA (2020). COVID-19: The Conjunction of Events Leading to the Coronavirus Pandemic and Lessons to Learn for Future Threats. Frontiers in Medicine.

[CR47] Frutos R, Serra-Cobo J, Chen T, Devaux CA (2020). COVID-19: Time to exonerate the pangolin from the transmission of SARS-CoV-2 to humans. Infection, Genetics and Evolution.

[CR48] Gerdts V, Zakhartchouk A (2017). Vaccines for porcine epidemic diarrhea virus and other swine coronaviruses. Veterinary Microbiology.

[CR49] Ghai RR, Carpenter A, Liew AY, Martin KB, Herring MK, Gerber SI, Hall AJ, Sleeman JM, VonDobschuetz S, Behravesh CB (2021). Animal Reservoirs and Hosts for Emerging Alphacoronaviruses and Betacoronaviruses. Emerg Infect Dis.

[CR50] Godoy MG, Kibenge MJT, Kibenge FSB (2021). SARS-CoV-2 transmission via aquatic food animal species or their products: A review. Aquaculture.

[CR51] Gortázar C, de la Fuente J (2020). COVID-19 is likely to impact animal health. Preventive Veterinary Medicine.

[CR52] Gossner C, Danielson N, Gervelmeyer A, Berthe F, Faye B, Kaasik Aaslav K, Adlhoch C, Zeller H, Penttinen P, Coulombier D (2016). Human–dromedary camel interactions and the risk of acquiring zoonotic Middle East respiratory syndrome coronavirus infection. Zoonoses and Public Health.

[CR53] Gray GC, Merchant JA (2018). Pigs, pathogens, and public health. The Lancet Infectious Diseases.

[CR54] Grgić H, Bruce Hunter D, Hunton P, Nagy É (2009). Vaccine efficacy against Ontario isolates of infectious bronchitis virus. Canadian Journal of Veterinary Research.

[CR55] Grubaugh ND, Hanage WP, Rasmussen AL (2020). Making sense of mutation: what D614G means for the COVID-19 pandemic remains unclear. Cell.

[CR56] Gruetzmacher K, Karesh WB, Amuasi JH, Arshad A, Farlow A, Gabrysch S, Jetzkowitz J, Lieberman S, Palmer C, Winkler A (2020). The Berlin principles on one health–Bridging global health and conservation. Science of the Total Environment.

[CR57] Halabowski D, Rzymski P (2020). Taking a lesson from the COVID-19 pandemic: Preventing the future outbreaks of viral zoonoses through a multi-faceted approach. Science of the Total Environment.

[CR58] Halfmann PJ, Hatta M, Chiba S, Maemura T, Fan S, Takeda M, Kinoshita N, Hattori S, Sakai-Tagawa Y, Iwatsuki-Horimoto K (2020). Transmission of SARS-CoV-2 in domestic cats. New England Journal of Medicine.

[CR59] Hammer AS, Quaade ML, Rasmussen TB, Fonager J, Rasmussen M, Mundbjerg K, Lohse L, Strandbygaard B, Jørgensen CS, Alfaro-Núñez A (2021). SARS-CoV-2 transmission between mink (Neovison vison) and humans, Denmark. Emerging Infectious Diseases.

[CR60] He S, Han J, Lichtfouse E (2021). Backward transmission of COVID-19 from humans to animals may propagate reinfections and induce vaccine failure.

[CR61] Heywood AE, Macintyre CR (2020). Elimination of COVID-19: what would it look like and is it possible?. The Lancet Infectious Diseases.

[CR62] Hosie MJ, Hofmann-Lehmann R, Hartmann K, Egberink H, Truyen U, Addie DD, Belák S, Boucraut-Baralon C, Frymus T, Lloret A (2021). Anthropogenic infection of cats during the 2020 COVID-19 pandemic. Viruses.

[CR63] Hulme PE (2020). One Biosecurity: a unified concept to integrate human, animal, plant, and environmental health. Emerging Topics in Life Sciences.

[CR64] Jackwood MW, Hilt DA, Sellers HS, Williams SM, Lasher HN (2010). Rapid heat-treatment attenuation of infectious bronchitis virus. Avian Pathology.

[CR65] Ji W, Li X, Chen S, Ren L (2021). Transmission of SARS-CoV-2 via fomite, especially cold chain, should not be ignored. Proceedings of the National Academy of Sciences of the United States of America.

[CR66] Johnson CK, Hitchens PL, Pandit PS, Rushmore J, Evans TS, Young CCW, Doyle MM (2020). Global shifts in mammalian population trends reveal key predictors of virus spillover risk. Proceedings of the Royal Society B.

[CR67] Jordan B (2017). Vaccination against infectious bronchitis virus: a continuous challenge. Veterinary Microbiology.

[CR68] Keesing F, Belden LK, Daszak P, Dobson A, Harvell CD, Holt RD, Hudson P, Jolles A, Jones KE, Mitchell CE (2010). Impacts of biodiversity on the emergence and transmission of infectious diseases. Nature.

[CR69] Khamassi Khbou M, Jedidi MD, Zaafouri FB, Benzarti M (2021). Coronaviruses in farm animals: Epidemiology and public health implications. Veterinary Medicine and Science.

[CR70] Kim H, Lee Y, Kang SC, Han BK, Choi KM (2016). Recent vaccine technology in industrial animals. Clinical and Experimental Vaccine Research.

[CR71] Kim Y, MY, SM Goyal, MCJ Cheeran, and M Torremorell.  (2017). Evaluation of biosecurity measures to prevent indirect transmission of porcine epidemic diarrhea virus. BMC Veterinary Research.

[CR72] Kock MD (1996). Wildlife, people and development: veterinary contributions to wildlife health and resource management in Africa. Tropical Animal Health and Production.

[CR73] Koopmans M (2021). SARS-CoV-2 and the human-animal interface: outbreaks on mink farms. The Lancet Infectious Diseases.

[CR74] Koyama T, Weeraratne D, Snowdon JL, Parida L (2020). Emergence of drift variants that may affect COVID-19 vaccine development and antibody treatment. Pathogens.

[CR75] Kulkarni DD, Tosh C, Venkatesh G, Senthil D, Kumar.  (2013). Nipah virus infection: current scenario. Indian Journal of Virology : an Official Organ of Indian Virological Society.

[CR76] Kumar KS (2020) Covid-19 and Animal Health Concerns-A Veterinarian Perspective. *Journal homepage: *http://www.ijcmas.com, 9

[CR77] Lam TT-Y, Jia N, Zhang Y-W, Shum MH-H, Jiang J-F, Zhu H-C, Tong Y-G, Shi Y-X, Ni X-B, Liao Y-S (2020). Identifying SARS-CoV-2-related coronaviruses in Malayan pangolins. Nature.

[CR78] Latinne A, Saputro S, Kalengkongan J, Kowel CL, Gaghiwu L, Ransaleleh TA, Nangoy MJ, Wahyuni I, Kusumaningrum T, Safari D (2020). 'Characterizing and quantifying the wildlife trade network in Sulawesi. Indonesia', Global Ecology and Conservation.

[CR79] Lau SKP, Luk HKH, Wong ACP, Li KSM, Zhu L, He Z, Fung J, Chan TTY, Fung KSC, Woo PCY (2020). Possible bat origin of severe acute respiratory syndrome coronavirus 2. Emerging Infectious Diseases.

[CR80] Lawal N, Onoja AB (2020). The veterinary perspective of COVID-19. Sokoto Journal of Veterinary Sciences.

[CR81] Lee C (2015). Porcine epidemic diarrhea virus: an emerging and re-emerging epizootic swine virus. Virology Journal.

[CR82] Lee J, Hughes T, Lee M-H, Field H, Rovie-Ryan JJ, Sitam FT, Sipangkui S, Nathan SKSS, Ramirez D, Vijay Kumar S (2020) No evidence of coronaviruses or other potentially zoonotic viruses in Sunda pangolins (Manis javanica) entering the wildlife trade via Malaysia. *BioRxiv*10.1007/s10393-020-01503-x PMC7682123 33226526

[CR83] Legendre AM, Kuritz T, Galyon G, Baylor VM, Heidel RE (2017). Polyprenyl immunostimulant treatment of cats with presumptive non-effusive feline infectious peritonitis in a field study. Frontiers in Veterinary Science.

[CR84] Leopardi S, Priori P, Zecchin B, Zamperin G, Milani A, Tonon F, Giorgiutti M, Beato MS, De Benedictis P (2021). Interface between Bats and Pigs in Heavy Pig Production. Viruses.

[CR85] Leroy EM, Ar Gouilh M, Brugère-Picoux J (2020). The risk of SARS-CoV-2 transmission to pets and other wild and domestic animals strongly mandates a one-health strategy to control the COVID-19 pandemic. One Health..

[CR86] Li H-Y, Zhu G-J, Zhang Y-Z, Zhang L-B, Hagan EA, Martinez S, Chmura AA, Francisco L, Tai H, Miller M (2020). A qualitative study of zoonotic risk factors among rural communities in southern China. International Health.

[CR87] Li H, Mendelsohn E, Zong C, Zhang W, Hagan E, Wang N, Li S, Yan H, Huang H, Zhu G (2019) Biosafety and Health 10.1016/j.bsheal.2019.10.004 PMC7148670 32501444

[CR88] Looi LM, Chua KB (2007). Lessons from the Nipah virus outbreak in Malaysia. Malaysian Journal of Pathology.

[CR89] Mackenzie JS (2009) Global Outbreak Alert and Response Network (GOARN) and One Health: Nipah virus as a source of lessons learnt—CDC Presentation. In.: March

[CR90] Maclachlan NJ, Dubovi EJ (2010). Fenner's veterinary virology.

[CR91] Magouras I, Brookes VJ, Jori F, Martin A, Pfeiffer DU, Dürr S (2020). Emerging Zoonotic Diseases: Should We Rethink the Animal-Human Interface?. Frontiers in Veterinary Science.

[CR92] Manes C, Gollakner R, Capua I (2020) Could Mustelids spur COVID-19 into a panzootic? *Veterinaria italiana* 10.12834/VetIt.2375.13627.1 32909703

[CR93] Manyweathers J, Field H, Jordan D, Longnecker N, Agho K, Smith C, Taylor M (2017). Risk Mitigation of Emerging Zoonoses: Hendra Virus and Non-Vaccinating Horse Owners. Transboundary and Emerging Diseases.

[CR94] Martin MP, Wakenell PS, Woolcock P, B O'connor.  (2007). Evaluation of the effectiveness of two infectious bronchitis virus vaccine programs for preventing disease caused by a California IBV field isolate. Avian Diseases.

[CR95] McAloose D, Laverack M, Wang L, Killian ML, Caserta LC, Yuan F, Mitchell PK, Queen K, Mauldin MR, Cronk BD (2020) From people to Panthera: Natural SARS-CoV-2 infection in tigers and lions at the Bronx Zoo. *Mbio* 11 10.1128/mBio.02220-20 PMC7554670 33051368

[CR96] McNamara T, Richt JA, Glickman L (2020). A critical needs assessment for research in companion animals and livestock following the pandemic of COVID-19 in humans. Vector-Borne and Zoonotic Diseases.

[CR97] Mendez DH, Kelly J, Buttner P, Nowak M, Speare R (2014). Management of the slowly emerging zoonosis, Hendra virus, by private veterinarians in Queensland, Australia: a qualitative study. BMC Veterinary Research.

[CR98] Mihindukulasuriya KA, Wu G, Leger JS, Nordhausen RW, Wang D (2008). Identification of a novel coronavirus from a beluga whale by using a panviral microarray. Journal of Virology.

[CR99] Miłek J, Blicharz-Domańska K (2018). Coronaviruses in avian species–review with focus on epidemiology and diagnosis in wild birds. Journal of Veterinary Research.

[CR100] Molenaar RJ, Vreman S, Hakze-van RW, der Honing R, de Zwart J, Rond E Weesendorp, Smit LAM, Koopmans M, Bouwstra R, Stegeman A (2020). Clinical and pathological findings in SARS-CoV-2 disease outbreaks in farmed mink (Neovison vison). Veterinary Pathology.

[CR101] Morens DM, Breman JG, Calisher CH, Doherty PC, Hahn BH, Keusch GT, Kramer LD, LeDuc JW, Monath TP, Taubenberger JK (2020). The origin of COVID-19 and why it matters. The American Journal of Tropical Medicine and Hygiene.

[CR102] Munnink BBO, Sikkema RS, Nieuwenhuijse DF, Molenaar RJ, Munger E, Molenkamp R, van der Spek A, Tolsma P, Rietveld A, Brouwer M (2020) 'Jumping back and forth: anthropozoonotic and zoonotic transmission of SARS-CoV-2 on mink farms. *BioRxiv*

[CR103] Munnink BBO, Sikkema RS, Nieuwenhuijse DF, Molenaar RJ, Munger E, Molenkamp R, van der Spek A, Tolsma P, Rietveld A, Brouwer M (2021). Transmission of SARS-CoV-2 on mink farms between humans and mink and back to humans. Science.

[CR104] Murphy BG, Perron M, Murakami E, Bauer K, Park Y, Eckstrand C, Liepnieks M, Pedersen Niels C (2018). The nucleoside analog GS-441524 strongly inhibits feline infectious peritonitis (FIP) virus in tissue culture and experimental cat infection studies. Veterinary Microbiology.

[CR105] Murray GM, O'Neill RG, More SJ, McElroy MC, Earley B, Cassidy JP (2016). Evolving views on bovine respiratory disease: An appraisal of selected key pathogens–Part 1. The Veterinary Journal.

[CR106] Nahar N, Paul RC, Sultana R, Sumon SA, Banik KC, Abedin J, Asaduzzaman M, Garcia F, Zimicki S, Rahman M (2017). A controlled trial to reduce the risk of human Nipah virus exposure in Bangladesh. Ecohealth.

[CR107] Nuismer SL, Bull JJ (2020). Self-disseminating vaccines to suppress zoonoses. Nature Ecology & Evolution.

[CR108] Olival KJ, Cryan PM, Amman BR, Baric RS, Blehert DS, Brook CE, Calisher CH, Castle KT, Coleman JTH, Daszak P (2020). Possibility for reverse zoonotic transmission of SARS-CoV-2 to free-ranging wildlife: A case study of bats. PLoS Pathogens.

[CR109] Omrani AS, Shalhoub S (2015). Middle East respiratory syndrome coronavirus (MERS-CoV): what lessons can we learn?. Journal of Hospital Infection.

[CR110] Opriessnig T, Huang Y‐W (2020) Further information on possible animal sources for human COVID‐19. *Xenotransplantation*10.1111/xen.12651 PMC7536993 32978828

[CR111] Osburn B, Scott C, Gibbs P (2009). One world—one medicine—one health: emerging veterinary challenges and opportunities. Revue Scientifique Et Technique.

[CR112] Osterhaus ADME, Vanlangendonck C, Barbeschi M, Bruschke CJM, Christensen R, Daszak P, de Groot F, Doherty P, Drury P, Gmacz S (2020). Make science evolve into a One Health approach to improve health and security: a white paper. One Health Outlook.

[CR113] Pang X, Ren L, Wu S, Ma W, Yang J, Di L, Li J, Xiao Y, Kang L, Du S (2020). Cold-chain food contamination as the possible origin of Covid-19 resurgence in Beijing. National Science Review.

[CR114] Patrono LV, Samuni L, Corman VM, Nourifar L, Röthemeier C, Wittig RM, Drosten C, Calvignac-Spencer S, Leendertz FH (2018). Human coronavirus OC43 outbreak in wild chimpanzees, Côte d´ Ivoire, 2016. Emerging Microbes & Infections.

[CR115] Pedersen NC, Kim Y, Liu H, Galasiti Kankanamalage AC, Eckstrand C, Groutas WC, Bannasch M, Meadows JM, Chang K-O (2018). Efficacy of a 3C-like protease inhibitor in treating various forms of acquired feline infectious peritonitis. Journal of Feline Medicine and Surgery.

[CR116] Pedersen NC, Perron M, Bannasch M, Montgomery E, Murakami E, Liepnieks M, Liu H (2019). Efficacy and safety of the nucleoside analog GS-441524 for treatment of cats with naturally occurring feline infectious peritonitis. Journal of Feline Medicine and Surgery.

[CR117] Plante JA, Liu Y, Liu J, Xia H, Johnson BA, Lokugamage KG, Zhang X, Muruato AE, Zou J, Fontes-Garfias CR (2021). Spike mutation D614G alters SARS-CoV-2 fitness. Nature.

[CR118] Pratelli A (2007). Action of disinfectants on canine coronavirus replication in vitro. Zoonoses and Public Health.

[CR119] Pulliam JRC, Epstein JH, Dushoff J, Rahman SA, Bunning M, Jamaluddin AA, Hyatt AD, Field HE, Dobson AP, Daszak P (2012). Agricultural intensification, priming for persistence and the emergence of Nipah virus: a lethal bat-borne zoonosis. Journal of the Royal Society Interface.

[CR120] Rabinowitz P, Conti L (2013). Links among human health, animal health, and ecosystem health. Annu Rev Public Health.

[CR121] Ren M, Han Z, Zhao Y, Sun J, Liu S, Ma D (2020). Multiple recombination events between field and vaccine strains resulted in the emergence of a novel infectious bronchitis virus with decreased pathogenicity and altered replication capacity. Poultry Science.

[CR122] Riddell S, Goldie S, Hill A, Eagles D, Drew TW (2020). The effect of temperature on persistence of SARS-CoV-2 on common surfaces. Virology Journal.

[CR123] Robertson ID (2020). Disease control, prevention and on-farm biosecurity: the role of veterinary epidemiology. Engineering.

[CR124] Roche B, Garchitorena A, Guégan J-F, Arnal A, Roiz D, Morand S, Zambrana-Torrelio C, Suzán G, Daszak P (2020). Was the COVID-19 pandemic avoidable? A call for a “solution-oriented” approach in pathogen evolutionary ecology to prevent future outbreaks. Ecology Letters.

[CR125] Roe D, Dickman A, Kock R, Milner-Gulland EJ, Rihoy E (2020). Beyond banning wildlife trade: COVID-19, conservation and development. World Development.

[CR126] Sabir JSM, Lam TT-Y, Ahmed MMM, Li L, Shen Y, Abo-Aba SEM, Qureshi MI, Abu-Zeid M, Zhang Y, Khiyami MA (2016). Co-circulation of three camel coronavirus species and recombination of MERS-CoVs in Saudi Arabia. Science.

[CR127] Sanders JM, Monogue ML, Jodlowski TZ, Cutrell JB (2020). Pharmacologic treatments for coronavirus disease 2019 (COVID-19): a review. Jama.

[CR128] Scott FW (1999). Evaluation of risks and benefits associated with vaccination against coronavirus infections in cats. Advances in Veterinary Medicine.

[CR129] Shen X, Sabir JSM, Irwin DM, Shen Y (2019). Vaccine against Middle East respiratory syndrome coronavirus. The Lancet Infectious Diseases.

[CR130] Singla R, Mishra A, Joshi R, Jha S, Sharma AR, Upadhyay S, Sarma P, Prakash A, Medhi B (2020). Human animal interface of SARS-CoV-2 (COVID-19) transmission: a critical appraisal of scientific evidence. Veterinary Research Communications.

[CR131] Sit THC, Brackman CJ, Ip SM, Tam KWS, Law PYT, To EMW, Yu VYT, Sims LD, Tsang DNC, Chu DKW (2020). Infection of dogs with SARS-CoV-2. Nature.

[CR132] Sivasundaram S (2020). The Human, The Animal and the Prehistory of COVID-19*. Past & Present.

[CR133] Sofi OMU, Sofi TA, Bandroo G, Javid F, Nabi B, Sheikh AA (2021). Possible Collaboration of Veterinarians and Medicos over the Fight against Covid-19 with “One-Health Approach”. Virol Mycol.

[CR134] Stevenson GW, Hoang H, Schwartz KJ, Burrough ER, Sun D, Madson D, Cooper VL, Pillatzki A, Gauger P, Schmitt BJ (2013). Emergence of Porcine epidemic diarrhea virus in the United States: clinical signs, lesions, and viral genomic sequences. Journal of Veterinary Diagnostic Investigation.

[CR135] Stokstad M, Klem TB, Myrmel M, Oma VS, Toftaker I, Østerås O, Nødtvedt A (2020). Using biosecurity measures to combat respiratory disease in cattle: The Norwegian control program for bovine respiratory syncytial virus and bovine coronavirus. Frontiers in Veterinary Science.

[CR136] Stower H (2020). Lopinavir–ritonavir in severe COVID-19. Nature Medicine.

[CR137] Summers J, Cheng H-Y, Lin H-H, Barnard LT, Kvalsvig A, Wilson N, Baker MG (2020) Potential lessons from the Taiwan and New Zealand health responses to the COVID-19 pandemic. *The Lancet Regional Health-Western Pacific* 100044 10.1016/j.lanwpc.2020.100044 PMC7577184 34013216

[CR138] Sumption K, Knight-Jones TJD, McLaws M, Paton DJ (2020). Parallels, differences and lessons: a comparison of the management of foot-and-mouth disease and COVID-19 using UK 2001/2020 as points of reference. Proceedings of the Royal Society B.

[CR139] Temmam S, Barbarino A, Maso D, Behillil S, Enouf V, Huon C, Jaraud A, Chevallier L, Backovic M, Pérot P (2020). Absence of SARS-CoV-2 infection in cats and dogs in close contact with a cluster of COVID-19 patients in a veterinary campus. One Health.

[CR140] Tizard IR (2020). Vaccination against coronaviruses in domestic animals. Vaccine.

[CR141] Tun HM, Cai Z, Khafipour E (2016). Monitoring survivability and infectivity of porcine epidemic diarrhea virus (PEDv) in the infected on-farm earthen manure storages (EMS). Frontiers in Microbiology.

[CR142] van Roon A, Maas M, Toale D, Tafro N, van der Giessen J (2019). Live exotic animals legally and illegally imported via the main Dutch airport and considerations for public health. PloS One.

[CR143] Vennema H, De Groot RJ, Harbour DA, Dalderup M, Gruffydd-Jones T, Horzinek MC, Spaan WJ (1990). Early death after feline infectious peritonitis virus challenge due to recombinant vaccinia virus immunization. Journal of Virology.

[CR144] Vlasova, AN, Wang Q, Jung K, Langel SN, Malik YS, Saif LJ (2020) Porcine coronaviruses. In *Emerging and Transboundary Animal Viruses* (Springer)

[CR145] Vlasova AN, Diaz A, Damtie D, Xiu L, Toh T-H, Lee JS-Y, Saif LJ, Gray GC (2021) Novel Canine Coronavirus Isolated from a Hospitalized Pneumonia Patient, East Malaysia. *Clinical Infectious Diseases*10.1093/cid/ciab456 PMC8194511 34013321

[CR146] Walsh MG, Wiethoelter A, Haseeb MA (2017). The impact of human population pressure on flying fox niches and the potential consequences for Hendra virus spillover. Scientific Reports.

[CR147] Wang Q, Vlasova AN, Kenney SP, Saif LJ (2019). Emerging and re-emerging coronaviruses in pigs. Current Opinion in Virology.

[CR148] Wardeh M, Baylis M, Blagrove MSC (2021). Predicting mammalian hosts in which novel coronaviruses can be generated. Nature Communications.

[CR149] Watsa M (2020). Rigorous wildlife disease surveillance. Science.

[CR150] Woo PCY, Lau SKP, Lam CSF, Tsang AKL, Hui S-W, Fan RYY, Martelli P, Yuen K-Y (2014). Discovery of a novel bottlenose dolphin coronavirus reveals a distinct species of marine mammal coronavirus in Gammacoronavirus. Journal of Virology.

[CR151] Woo PCY, Lau SKP, Wernery U, Wong EYM, Tsang AKL, Johnson B, Yip CCY, Lau CCY, Sivakumar S, Cai J-P (2014). Novel betacoronavirus in dromedaries of the Middle East, 2013. Emerging Infectious Diseases.

[CR152] Wünschmann A, Frank R, Pomeroy K, Kapil S (2002). Enteric coronavirus infection in a juvenile dromedary (Camelus dromedarius). Journal of Veterinary Diagnostic Investigation.

[CR153] Xiao K, Zhai J, Feng Y, Zhou N, Zhang X, Zou J-J, Li N, Guo Y, Li X, Shen X, Zhang Z, Shu F, Huang W, Li Y, Zhang Z, Chen R-A, Wu Y-J, Peng S-M, Huang M, Xie W-J, Cai Q-H, Hou F-H, Chen W, Xiao L, Shen Y (2020). Isolation of SARS-CoV-2-related coronavirus from Malayan pangolins. Nature.

[CR154] Xiao Y, Meng Q, Yin X, Guan Y, Liu Y, Li C, Wang M, Liu G, Tong T, Wang L-F (2008). Pathological changes in masked palm civets experimentally infected by severe acute respiratory syndrome (SARS) coronavirus. Journal of Comparative Pathology.

[CR155] Ye Z-W, Yuan S, Yuen K-S, Fung S-Y, Chan C-P, Jin D-Y (2020). Zoonotic origins of human coronaviruses. International Journal of Biological Sciences.

[CR156] Zhang T, Wu Q, Zhang Z (2020). 'Probable pangolin origin of SARS-CoV-2 associated with the COVID-19 outbreak. Current Biology.

[CR157] Zhou P, Fan H, Lan T, Yang X-L, Shi W-F, Zhang W, Zhu Y, Zhang Y-W, Xie Q-M, Mani S (2018). Fatal swine acute diarrhoea syndrome caused by an HKU2-related coronavirus of bat origin. Nature.

[CR158] Zhu S, Zimmerman D, Deem SL (2019). A review of zoonotic pathogens of dromedary camels. EcoHealth.

